# Histone methylation and vascular biology

**DOI:** 10.1186/s13148-020-00826-4

**Published:** 2020-02-18

**Authors:** Xiang Wei, Xin Yi, Xue-Hai Zhu, Ding-Sheng Jiang

**Affiliations:** 1 Division of Cardiothoracic and Vascular Surgery, Tongji Hospital, Tongji Medical College, Huazhong University of Science and Technology, 1095 Jiefang Ave, Wuhan, 430030 China; 2 Key Laboratory of Organ Transplantation, Ministry of Education, Wuhan, Hubei China; 3 NHC Key Laboratory of Organ Transplantation, Wuhan, Hubei China; 4 Key Laboratory of Organ Transplantation, Chinese Academy of Medical Sciences, Wuhan, Hubei China; 5 Department of Cardiology, Renmin Hospital of Wuhan University, Wuhan, Hubei China

**Keywords:** Histone methylation, Histone methyltransferase, Demethylase, Atherosclerosis, Intimal hyperplasia, Aortic dissection/aneurysm, Pulmonary arterial hypertension, Diabetic angiopathy, Cancer angiogenesis

## Abstract

The vasculature not only transports oxygenated blood, metabolites, and waste products but also serves as a conduit for hormonal communication between distant tissues. Therefore, it is important to maintain homeostasis within the vasculature. Recent studies have greatly expanded our understanding of the regulation of vasculature development and vascular-related diseases at the epigenetic level, including by protein posttranslational modifications, DNA methylation, and noncoding RNAs. Integrating epigenetic mechanisms into the pathophysiologic conceptualization of complex and multifactorial vascular-related diseases may provide promising therapeutic approaches. Several reviews have presented detailed discussions of epigenetic mechanisms not including histone methylation in vascular biology. In this review, we primarily discuss histone methylation in vascular development and maturity, and in vascular diseases.

The vasculature, which consists of arterial, venous, and interconnecting capillary beds, is formed through vasculogenesis or angiogenesis during embryogenesis. The walls of the vessels are composed of endothelial cells, mural cells, and the extracellular matrix (ECM). The origin, number, type, and organization of mural cells depend on the location of the vessel and its function. For example, the smooth muscle cells (SMCs) of the ascending and arch portions of the aorta originate from the neural crest, while the SMCs of the descending thoracic aorta are contributed by somite-derived cells [1]. The vasculature, a highly branched, tree-like, tubular network, not only transports oxygenated blood, metabolites, and waste products but also serves as a conduit for hormonal communication between distant tissues. Furthermore, the vasculature facilitates rapid deployment of immune responses to distal sites within the body [2]. Maintaining vascular biologic homeostasis is essential for the body; once this balance is disrupted, the vasculature will suffer from dysplasia or diseases, such as angiodysplasia [3], aortic aneurysm/dissection [4], atherosclerosis [5, 6], pulmonary arterial hypertension [7], diabetic angiopathy [8], or arteritis [9]. Multiple mechanisms are involved in the shift from the physiological status to the pathological state of the vasculature. Among them, epigenetic mechanisms (e.g., posttranslational modification, RNA methylation, DNA methylation, and miRNA) play an indispensable role during these processes [10, 11]. Several published reviews have summarized epigenetic regulation in vascular biology; in particular, noncoding RNAs, DNA methylation, and protein acetylation and phosphorylation have been widelydiscussed [12–14]. In recent years, m^6^A RNA methylation has emerged as anew research field, but the functions of m^6^A RNA methylation in vascular development and vascular diseases remain to be revealed. Incontrast, histone methylation has been investigated extensively in vascular biology after the discoveries of the first histone methyltransferase (HMT) in 2000 and the first histone demethylase in 2004 [15, 16]. Therefore, in the present review, we focus only on histone methylation and systematically summarize the research on the roles of histone methylation and mechanisms by which it is involved in vascular development and diseases.

## Histone methylation

**Fig. 1:**
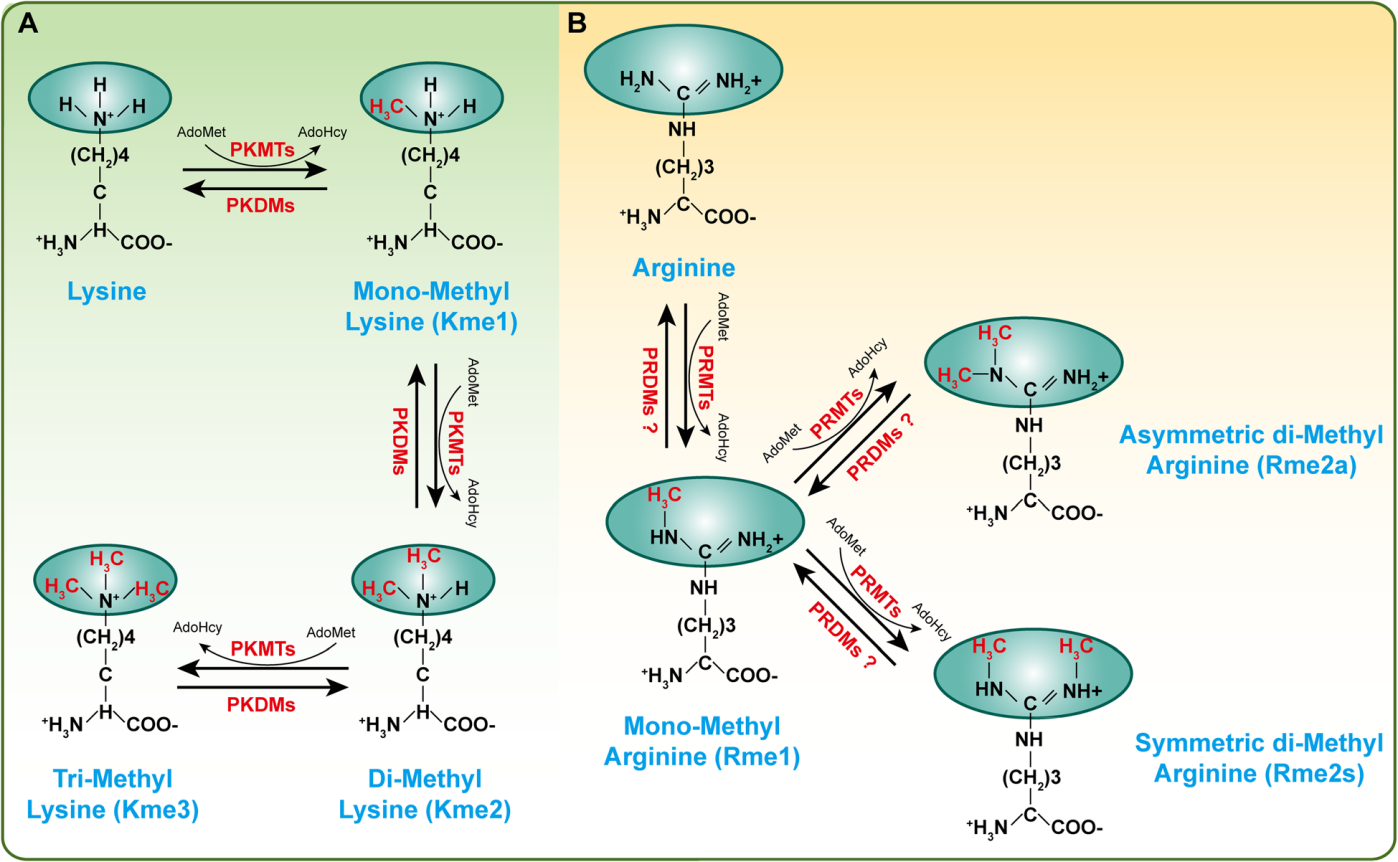
A schematic diagram of histone methylation on lysine or arginine residues. Protein can be methylated by methyltransferases and S-adenosyl-l-methionine (AdoMet) is used as the primary methylgroup donor, while these modifications are reversible and can be erased by demethylases. **a** Protein lysine methyltransferases (PKMTs) catalyze monomethylation (Kme1), dimethylation (Kme2) and trimethylation (Kme3) of proteins on the ε-amine group of lysine. **b** Protein arginine methyltransferases (PRMTs) methylate the ω-amino group of arginine residues, resulting in either monomethylated (Rme1) or symmetric (Rme2s) or asymmetric (Rme2a) dimethylation. *PKDMs* protein lysine demethylases, *PRDMs* protein arginine demethylases

Histone methylation, a reversible posttranslational modification, is written by HMTs and erased by histone demethylases (HDMTs) [17]. To date, two main types of histone methylation have been identified: methylation on lysine and arginine residues. Correspondingly, HMTs have been divided into two categories: protein lysine methyltransferases (PKMTs) and protein arginine methyltransferases (PRMTs) [18, 19]. The ε-amine group of lysine can be marked with monomethylation (me1), dimethylation (me2), and trimethylation (me3) by suppressor of variegation, enhancer of Zeste, Trithorax (SET) domain-containing PKMTs or non-SET-domain-containing PKMTs [18, 20, 21] (Fig. 1a). In contrast, arginine is methylated by PRMTs at ω-amino groups, which appeared as monomethylation (MMA, Rme1), symmetric dimethylarginine (SDMA, Rme2s), and asymmetric dimethylarginine (ADMA, Rme2a) (Fig. 1b) [22]. S-Adenosyl-l-methionine (AdoMet), the primary methyl group donor, interacts with PKMTs or PRMTs to transfer methyl groups to the lysine or arginine residues (Fig. 1) [23]. A variety of substrates can be methylated by HMTs, with canonical substrates being histones, such as H3K27, H3K4, H3K9, H4K20, and H3R17 [24–27]. However, with further research, an increasing number of nonhistone proteins (e.g., p53, Rb, and Hsp90) have been found to be methylated by HMTs [28, 29]. Methylation on nonhistone proteins is associated with other post-translational modifications (PTMs), such as phosphorylation and acetylation, which affects the activity or stability of proteins [30–32]. In recent years, many studies have revealed that histone methylation is involved in and indispensable for the development of a variety of vascular diseases. In this review, we discuss the role of histone methylation on vascular development and maturity, atherosclerosis and vascular intimal hyperplasia, acute thoracic aortic syndromes and aortic aneurysms, pulmonary arterial hypertension, diabetic angiopathy, endothelial dysfunction, and other forms of vasculopathy.

## Histone methylation in vascular development and maturity

Defects in placental vascular development cause embryonic death and abnormal organogenesis, negatively affect fetal growth, or confer a higher risk of disease during postnatal life [33]. Vascular remodeling is an important pregnancy-associated adaptation in hemochorial placentation, and the most common cause of placental dysfunction is the failure of vascular remodeling by extravillous trophoblast [34]. As reported by Rodesch et al. in 1992, they found that relatively hypoxic environment within the intervillous space of placenta (varies between 2 and 8%) than endometrial oxygen tension during early implantation [35, 36]. This environment is thought to facilitate the villous capillary network continued sprouting and remodeling throughout gestation [37]. The HIF signaling is a classic oxygen-sensitive pathway to regulate angiogenesis under hypoxic environments. Hypoxia activates *Hif*-dependent expression of lysine demethylase 3A (*Kdm3a*) which demethylates H3K9 to accelerate *Mmp12* expression to facilitate trophoblast invasion and uterine vascular remodeling [38].

**Fig. 2:**
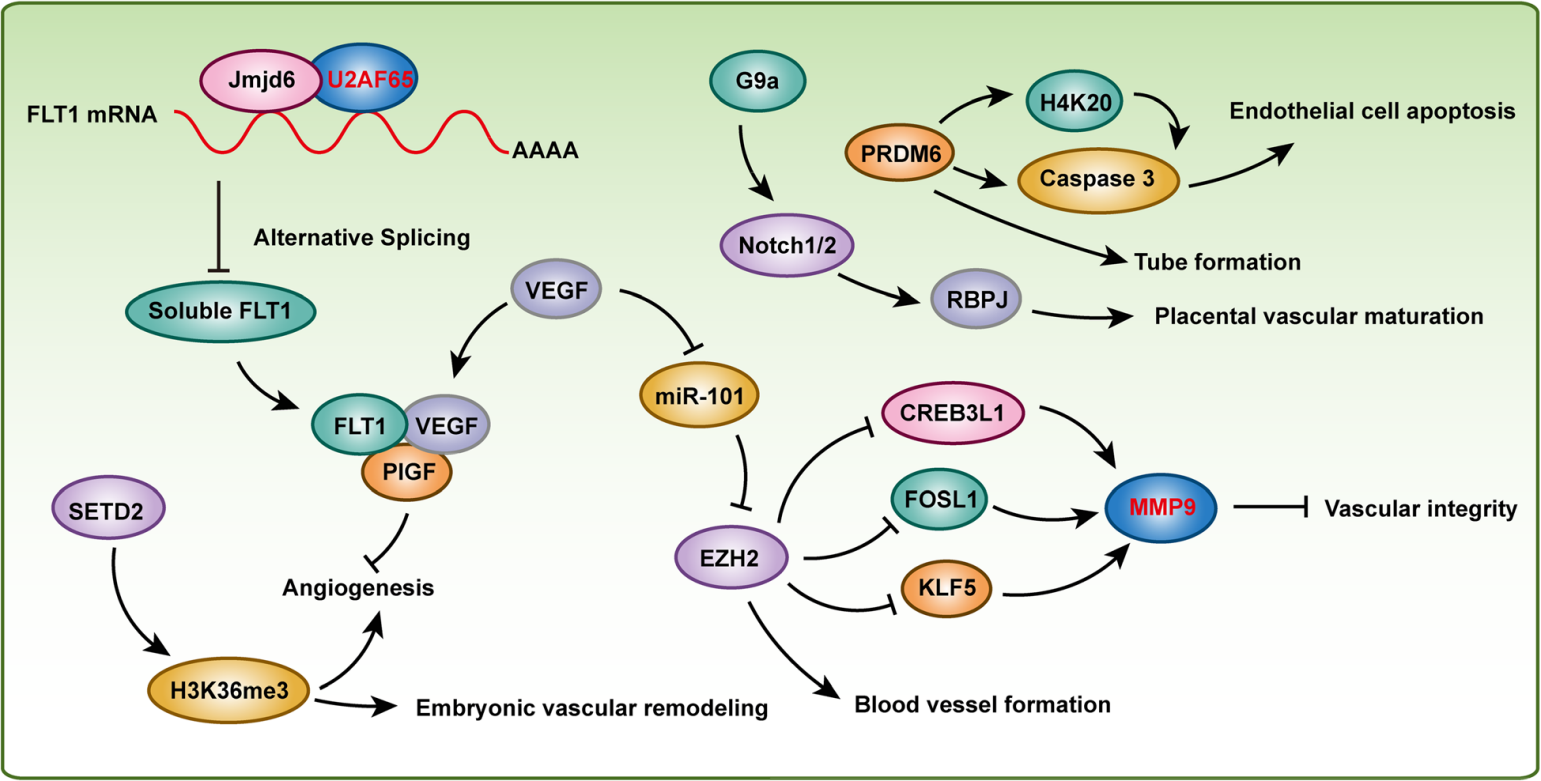
Histone methylation regulates vascular development and maturity. Histone arginine demethylase Jmjd6 and histone methyltransferases SETD2, EZH2, G9a, and PRDM6 are involved in vascular development and maturity

In mice in which the *Flk1* (also known as *Vegfr2*) gene was targeted for disruption, the absence of both endothelial and hematopoietic development was detected, and the mice died in utero on E8.0-E9.0, indicating that *Flk1*was required in the earliest stages of hematovascular development [39]. Histone-lysine *N*-methyltransferase *Prdm6* is enriched in *Flk1*(+) hematovascular precursor cells [40]. In mouse embryonic endothelial cells, overexpression of *Prdm6* induced apoptosis by activating caspase-3 and inducing G1 arrest and resulted in inhibited tube formation, which indicated that *Prdm6* may play a role in vascular cell precursor differentiation and survival [40]. *Flt1* (also known as *Vegfr1*), an important paralog of *Flk1*, was reported to be regulated by histone arginine demethylase *Jmjd6* which controlled angiogenic sprouting [41]. Jmjd6 interacted with splicing factor *U2af65* to alter the splicing of *Flt1*, affecting the levels of the soluble form of *Flt1*, which was subsequently bound to *Vegf* and placental growth factor (*Plgf*) to regulate angiogenesis [41]. VEGF treatment inhibited miR-101 expression in endothelial cells, and miR-101 targeted *Ezh2*, which methylated histone H3 lysine 27 (H3K27), suppressing gene expression. Furthermore, systemic administration of DZNep to inhibit *Ezh2* reduced the number of blood vessels in a subcutaneous glioblastoma mouse model [42]. In addition, *Ezh2* inhibited *Creb3l1*, *Fosl1*, *Klf5*, and *Mmp9* in endothelial cells to maintain the integrity of the developing vasculature [43]. MMP9 was also elevated significantly in blood samples from acute aortic dissection (AAD) patients, and the incidence of AAD was reduced significantly, by 40%, following the administration of an MMP inhibitor and was almost completely blocked in *Mmp9*^−/−^ mice [44]. More importantly, recent results from our studies demonstrated that *Ezh2* was involved in AAD by inhibiting the autophagic cell death that was regulated by the *Atg5*, *Atg7*, and *Mek1/2-Erk1/2* signaling pathway [24]. Histone methyltransferase G9a was reported to activate Notch pathway effectors (e.g., *Rbpj*) to control placental vascular maturation, and G9a and RBPJ were downregulated in human placentae from intrauterine growth restriction-affected pregnancies [33]. Given that the expression of Jagged1, a ligand involved in Notch signaling, was linked to increased circulating plasma VEGF in giant cell arteritis patient blood vessels, VEGF enhanced Jagged1 expression and vessel wall inflammation in mice which were implanted with patient peripheral blood mononuclear cells and human arteries [45]. Furthermore, Spuul et al. demonstrated that VEGF/Notch signaling regulates the formation of functional podosomes in endothelial cells to promote retinal neovascularization [46]. However, how histone methylation and its corresponding HMTs or HDMTs cooperates with VEGF/Notch signaling to regulate vascular development and maturity need further research. In addition, HYPB (also known as SETD2 and KMT3A) is a histone H3 lysine 36 (H3K36)-specific methyltransferase [27]. Homozygous disruption of *Hypb* resulted in embryonic lethality at E10.5-E11.5 due to severe vascular defects in the embryo, yolk sac, and placenta that was mediated by impaired H3K36 trimethylation but not monomethylation or dimethylation [3]. In early mammalian erythropoiesis, histone methyltransferase *Dot1l* plays a critical role in controlling the number of circulating erythroid and myeloid cells, as indicated by *Dot1l*-mutant mice that developed more slowly and died between E10.5 and E13.5, displaying profound anemia, which was especially apparent in the small vessels of the yolk sac. These effects were induced by inhibiting *Gata2* expression while enhancing PU.1 levels [47]. The findings from these aforementioned studies indicate that histone methylation plays an essential role in vascular development and maturity (Fig. 2). However, more investigation is needed to uncover whether other HMTs or HDMTs regulate angiogenesis, and more importantly, additional vascular system-specific HMT- and HDMT-knockout animal models should be used to interpret HMT and HDMT function in vascular development. In addition, ascertaining whether nonhistone proteins take part in these biological processes would be a valuable undertaking.

## Histone methylation in atherosclerosis and vascular intimal hyperplasia

**Fig. 3:**
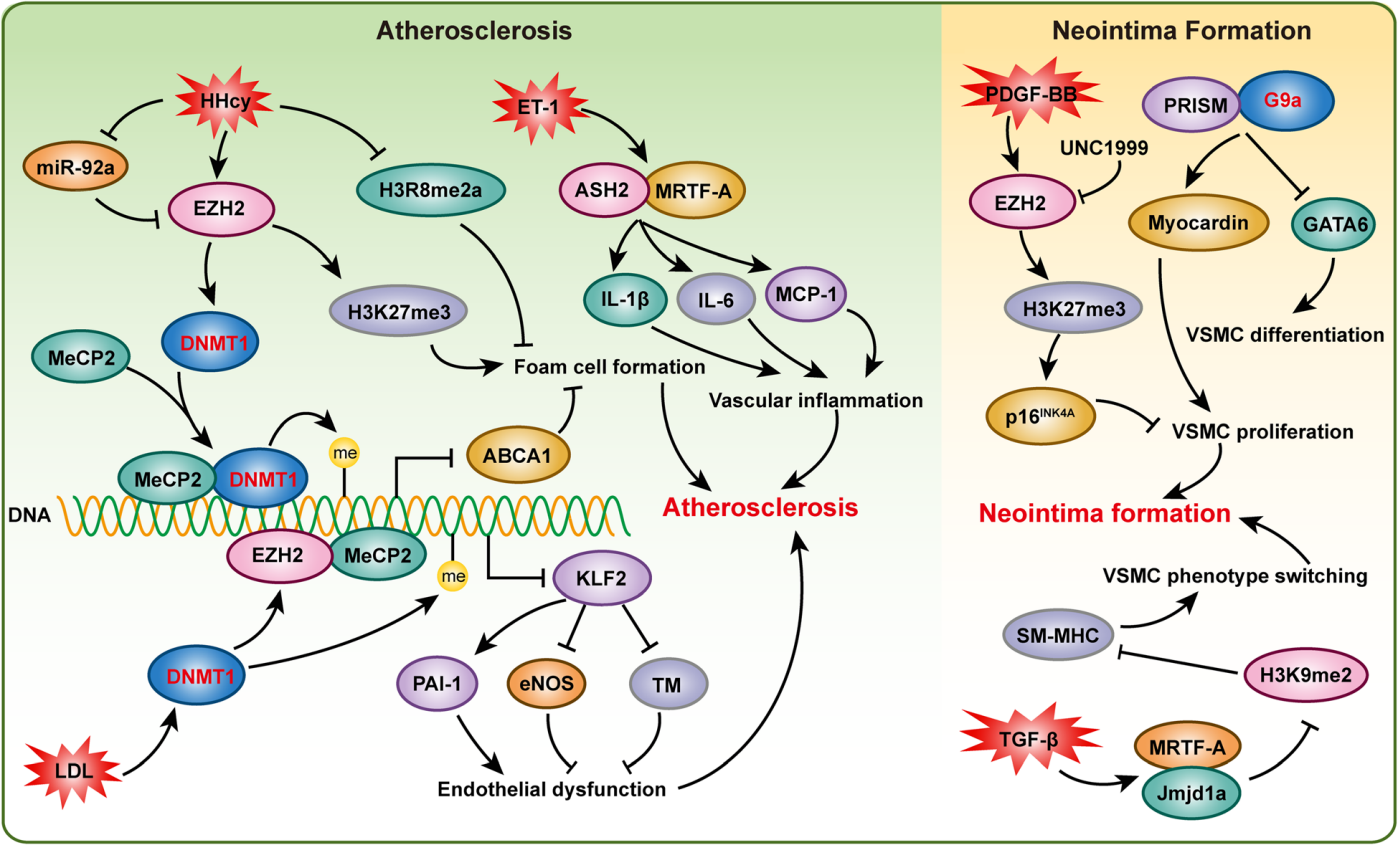
Histone methylation is critical for atherosclerosis and neointima formation. Histone methyltransferases EZH2 and ASH2 participate in atherosclerosis via regulating endothelial dysfunction, foam cell formation, and vascular inflammation, respectively. Histone methyltransferases EZH2 and G9a and demethylase Jmjd1A are involved in neointima formation by affecting vascular smooth muscle cell (VSMC) proliferation, differentiation and phenotype switching

Atherosclerosis, one of the primary causes of cardiovascular death worldwide, is initiated by endothelial dysfunction and lipid accumulation [5, 48], and it is characterized by fibrotic cell proliferation, chronic inflammation, lipid accumulation, and immune disorder in the vessel wall [49]. Vascular SMCs have been found to contribute to atherosclerotic plaque formation through proliferation, migration, and apoptosis, and they are involved in inflammation, extracellular matrix synthesis, and foam cell formation through cholesterol uptake [50]. Vulnerable plaques are prone to rupture after the atheromatous plaques develop into an advanced stage, which leads to acute cardiovascular events, including ischemic stroke and myocardial infarction [49]. Although the research is still in its infancy, emerging evidence is elucidating the role of epigenetic mechanisms in atherosclerosis. In this review, we focus on discussing histone methylation in atherosclerosis (Fig. 3). For reviews on other epigenetic mechanisms, the reader is referred to a review by Xu et al. [49].

Several studies have investigated global histone methylation in human atherosclerotic plaques [6, 51, 52]. Greißel et al. demonstrated that global H3K9me2 and H3K27me2 were significantly decreased in atherosclerotic lesions, while comparable H3K4me2 levels were identified in atherosclerotic and healthy carotid arteries [6]. Interestingly, the immunohistochemistry results revealed increased H3K4me2 levels but decreased H3K9me2 levels in VSMCs, as well as reduced H3K9me2 and H3K27me2 levels in inflammatory cells. Paradoxically, the expression of the corresponding histone methyltransferases MLL2 and G9a was increased in advanced atherosclerosis compared to early atherosclerosis [6]. In addition, this research group also demonstrated that H3K4 methylation and H3K9 acetylation were significantly associated with the severity of atherosclerosis [52]. Similarly, Wierda et al. also demonstrated that the global level of H3K27me3 was reduced in vessels with advanced atherosclerotic plaques, but this reduction in H3K27me3 level was not accompanied by alterations in the corresponding histone methyltransferase EZH2 or demethylase JMJD3 [51]. These results indicated that H3K9 and H3K27 demethylation was critical for atherosclerotic plaque formation. *Ezh2* the methyltransferase corresponding to H3K27 promoted foam cell formation and the development of atherosclerosis in *ApoE*^−/−^ mice. Mechanistically, *Ezh2* induced DNA methyltransferase 1 (*Dnmt1*) expression, methyl CpG-binding protein-2 (MeCP2) recruitment, and the binding of *Dnmt1* and MeCP2 to the ATP-binding cassette transporter A1 (*Abca1*) promoter, thereby promoting *Abca1* gene DNA methylation, which inhibited *Abca1* expression and accelerated atherosclerosis [53]. Elevated low-density lipoprotein (LDL) levels are a major risk factor for atherosclerosis development. Increased LDL induces endothelial *Dnmt1* expression and DNA methyltransferase activity and stimulated binding of MeCP2 and EZH2, which resulted in myocyte enhancing factor-2 (MEF2) dissociation from the KLF2 promoter to suppress KLF2 expression in endothelial cells. Decreased KLF2 led to thrombomodulin and endothelial nitric oxide synthase (eNOS) expression suppression and to PAI-1 activation, which impaired endothelial function [54]. Hyperhomocysteinemia (HHcy) is another independent risk factor for atherosclerosis. After *ApoE*^−/−^ mice were challenged with a high-methionine diet for 16 weeks, the levels of *Ezh2* and H3K27me3 were increased in their aortas, which promoted the accumulation of total cholesterol and triglycerides in foam cells, and miR-92a inhibited this HHcy-mediated lipid metabolism disorders by targeting *Ezh2* [55]. These studies indicated that *Ezh2* and *Dnmt1* could form a positive feedback regulation fashion. On the one hand, they regulate the formation of foam cells by inhibiting ATP-binding cassette transporter A1 (ABCA1); on the other hand, they affect endothelial dysfunction by suppressing KLF2, and jointly promote the formation of atherosclerosis. It is also a model of the interconnection between histone methylation and DNA methylation. In an animal model of diet-induced HHcy, Esse et al. showed that severe HHcy disrupted global protein arginine methylation in a tissue-specific manner, especially the H3R8me2a mark, the level of which was profoundly decreased [56]. In addition, histone-arginine methyltransferase *Prmt4* and demethylase Jmjd6 participated with low-density lipoprotein receptor-related protein 6 (*Lrp6*) to promote arteriosclerotic calcification in diabetic *Ldlr*^−/−^ mice [57]. ASH2, a histone methyltransferase complex subunit, interacted with MRTF-A to transactivate pro-inflammatory genes in VSMCs in response to endothelin (ET-1) treatment [58].

Angioplasty and coronary artery bypass grafting are highly effective treatment for narrowed coronary arteries due to atherosclerosis. However, restenosis resulting from neointima hyperplasia after angioplasty greatly dampens the satisfactory prognosis of the atherosclerosis for patients [59]. Recent research advances have indicated that histone methylation is critical for regulating neointima hyperplasia (Fig. 3). For example, Liang et al. showed that PDGF-BB markedly increased H3K27me3 and *Ezh2* levels. Inhibition of *Ezh2*/*1* activity by UNC1999 significantly suppressed PDGF-BB-induced VSMC proliferation and neointima formation following wire-guided common carotid injury, which was mediated by increasing the transcription of the cyclin-dependent kinase inhibitor p16^INK4A^ [59]. Knockdown of *Jmjd1a* in primary rat aortic SMCs attenuated TGF-β-induced upregulation of endogenous SM myosin heavy chain expression by interacting with MRTF-A and regulating H3K9me2 levels to affect VSMC phenotype switching [60]. PRISM interacted with G9a histone methyltransferase and class I histone deacetylases to induce genes associated with the proliferative smooth muscle phenotype while repressing regulators of differentiation, including myocardin and GATA-6 in primary VSMCs [61]. H3K27me3 and H3K4me2 were reportedly involved in neointima formation by regulating *Myh11*, *Acta2*, *Cnn1*, and *Sm22* or *Vcam-1* expression [62, 63].

Although several kinds of HMTs and HDMTs were found to have changed expression levels during atherosclerosis or neointima formation, thereby affecting histone methylation levels, the potential roles of HMTs and HDMTs in atherosclerosis and neointima formation require further investigation. As many inhibitors targeting HMTs or HDMTs have been developed, with some in ongoing clinical trials for treating cancer, it is urgent to verify whether these inhibitors have the potential to reverse atherosclerosis or neointima formation in the near future.

## Histone methylation in acute thoracic aortic syndromes and aortic aneurysm

According to 2014 ESC guidelines on the diagnosis and treatment of aortic diseases, acute thoracic aortic syndromes (AASs) which include intramural hematoma (IMH), penetrating aortic ulcer (PAU), aortic dissection (AD), and thoracic aortic rupture are defined as emergency conditions with similar clinical characteristics involving the aorta [64]. Among them, AD is the disease that has been extensively investigated. AD is a life-threatening disease with an incidence of six per hundred thousand persons per year [65]. Furthermore, 50% of patients with acute type A AD who do not receive surgery die within the first 48 h of the event [64]. The pathological features of AD are characterized by an enlarged and degenerative medial layer, vascular smooth muscle cell (VSMC) loss or dysfunction, proteoglycan accumulation, and collagen and elastic fiber cross-linked disorder and fragmentation [66]. Our recent results demonstrated that EZH2, a methyltransferase for H3K27 dimethylation and trimethylation, was downregulated in the aortic wall of patients with AD compared with the levels in the normal controls [24]. Most importantly, EZH2 negatively regulated autophagosome formation by inhibiting ATG5 and ATG7 expression and the MEK1/2-ERK1/2 signaling pathway to prevent autophagic death of VSMCs. In addition, we also found that the protein levels of H3K9me2 and H3K23me1 were upregulated, while H4K20me2 was downregulated in the aorta samples of AD patients [67]. For abdominal aortic aneurysm (AAA), Jones et al. identified four new AAA-specific risk loci, including 1q32.3 (SMYD2), 13q12.11 (LINC00540), 20q13.12 (near PCIF1/MMP9/ZNF335), and 21q22.2 (ERG), via a meta-analysis of 6 genome-wide-associated study data sets and a validation study with a total of 10,204 cases and 107,766 controls [68]. Furthermore, Toghill et al. revealed that, in aortic tissues of AAA patients, the SMYD2 promoter was hypo-methylated and SMYD2 was downregulated compared to the methylation and expression levels of the respective controls [69]. These two related studies highlight the role of SMYD2 in AAA, but further investigation is needed to uncover its exact role and mechanisms. In addition, in human thoracic aortic aneurysms (TAAs), SMAD2 was upregulated, compared the level in normal aortas, and H3K9/14 acetylation and H3K4 methylation were involved in SMAD2 overexpression in TAAs [70].

Hypertension is identified as the most common risk factor associated with AD, as it was observed in 65–75% of the individuals with AD [64, 71]. Thus, the prevention and control of hypertension are critical ways to prevent and treat AD. It is well known that renin-angiotensin-aldosterone system (RAAS) dysregulation plays a crucial role in the development of hypertension; thus, the epigenetic regulation of RAAS-regulated genes has been extensively studied in hypertensive models [72, 73]. For example, in the aortas of spontaneously hypertensive rats (SHRs), enrichment of H3K4me3 but a decrease in H3K9me2 level was found at the angiotensin-converting enzyme 1 (*Ace1*) promoter, which is associated with *Ace1* upregulation [74]. Downregulation of the hydroxysteroid dehydrogenase-11β2 enzyme (*Hsd11b2*), a gene related to renal sodium balance, was associated with a decrease in H3K36me3 in SHRs [75]. Furthermore, higher levels of H4ac and H3K4me3, but lower levels of H3K27me3 and H3K9me3 at atrial natriuretic peptide (*Anp*) and brain natriuretic peptide (*Bnp*) gene promoters accelerated *Anp* and *Bnp* expression to regulate heart damage in the SHRs [75, 76].

The eNOS (also known as NOS3), constitutively expressed in vascular endothelial cells, plays a key role in vascular wall homeostasis and the regulation of vasomotor tone [77]. eNOS is critical for most vasoprotective molecule nitric oxide production, and vascular nitric oxide dilates all types of blood vessels by stimulating soluble guanylyl cyclase and increasing cyclic guanosine monophosphate (cGMP) levels in VSMCs [78]. In endothelial cells, H3K9ac, H4K12ac, H3K4me2, and H3K4me3 are enriched at the eNOS proximal promoter to regulate the basal expression of eNOS [77]. Lysine-specific demethylase-1 (LSD1) demethylates H3K4 and H3K9 to alter gene transcription. Heterozygous *Lsd1*-knockout mice (*Lsd1*^+/−^) had higher blood pressure than wild-type (WT) mice on a liberal salt diet but not on a salt-restricted diet [79]. In *Lsd1*^+/−^ mice, RAAS was suppressed, as shown by plasma renin activity and plasma levels and urinary excretion of aldosterone wad lower in *Lsd1*^+/−^ mice than in WT mice. Furthermore, decreased eNOS and guanylate cyclase expression indicated enhanced vascular contraction and reduced relaxation via the NO-cGMP pathway in the *Lsd1*^+/−^ mice on a liberal salt diet [79]. Endothelin-1, a potent vasoconstrictor derived from vascular endothelium, was induced by angiotensin II, which was accompanied by the accumulation of H3K4me3 on its promoter [80]. Under angiotensin II treatment, Suv, Ez, and Trithorax domain 1 (*Set1*), a histone H3K4 tri-methyltransferase, was recruited to the promoter of endothelin-1 by activating protein 1 (*Ap1*) to methylate H3K4, and in synergy with *Ap1*, to activate endothelin-1 transcription. Increased endothelin-1 expression resulted in vasoconstriction and elevated blood pressure, thereby contributing to angiotensin II-induced cardiac hypertrophy [80].

**Fig. 4:**
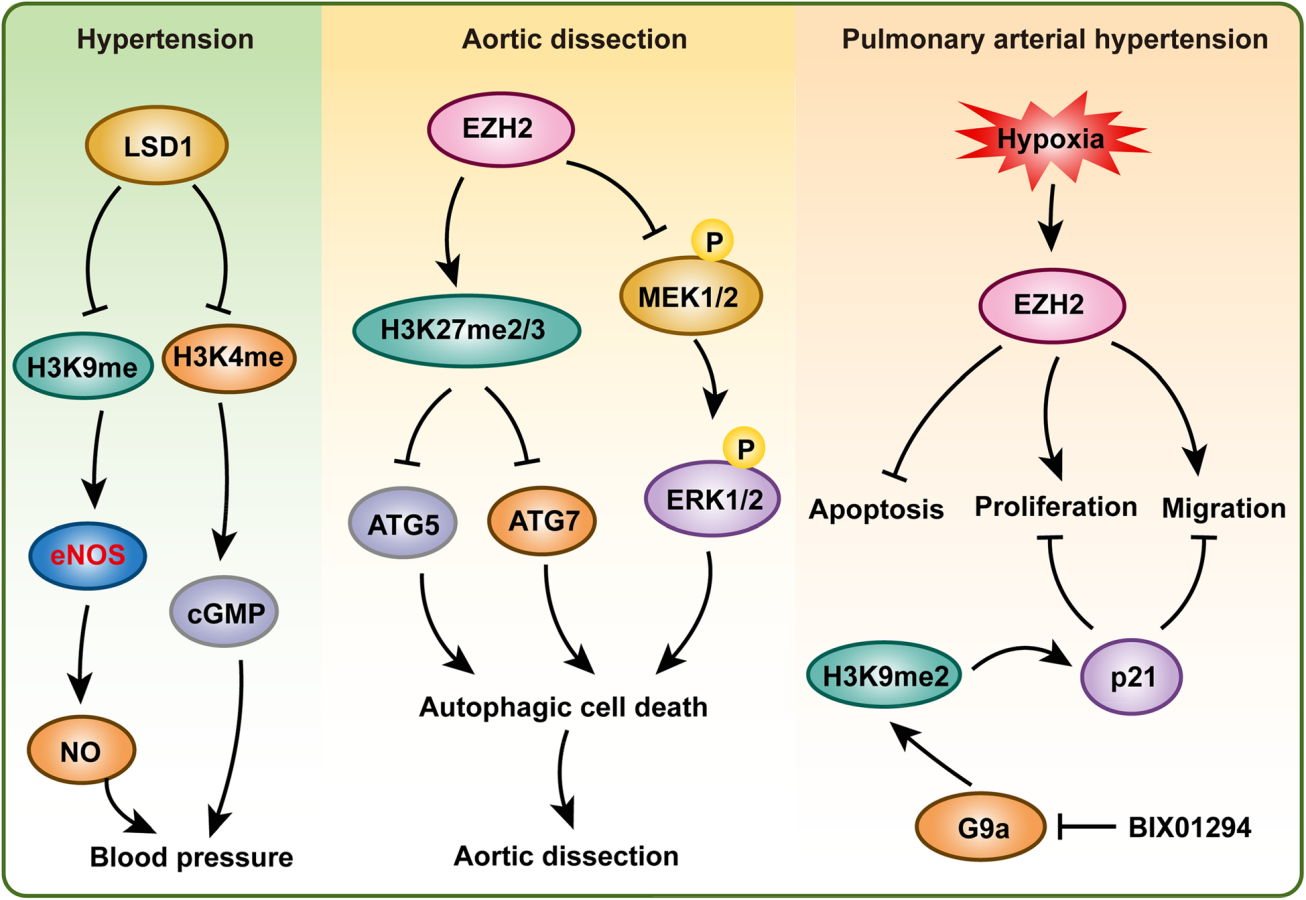
Histone methylation plays a role in hypertension, aortic dissection, and pulmonary arterial hypertension. Histone demethylase LSD1 was reported to regulate blood pressure. EZH2 inhibits autophagic death of VSMC to suppress aortic dissection by regulating ATG5 and ATG7 expression and MEK-ERK1/2 signaling pathway. In addition, EZH2 and G9a play a critical role in pulmonary arterial smooth muscle cells to affect pulmonary arterial hypertension

These results indicate that histone methylation is critical for AD, AAA, and TAA formation and VSMC survival, as well as being a risk factor hypertension (Fig. 4). However, the importance of histone methylation in aortic dissection has obviously been underrated, and more attention should be paid to this research field.

## Histone methylation in pulmonary arterial hypertension

Pulmonary hypertension (PH) is defined as a resting mean pulmonary artery pressure (mPAP) greater than or equal to 25 mmHg [81]. Pulmonary arterial hypertension (PAH) should meet the following criteria: pulmonary capillary wedge pressure (PCWP) that is below 15 mmHg, PVR ≥ 3 Wood units, and mPAP ≥ 25 mmHg, in the absence of more prevalent causes of pulmonary hypertension, such as chronic lung disease, left heart disease, or venous thromboembolism [81, 82]. The incidence of PAH ranges from 2 to 7.6 cases per million adults per year and is four-fold higher in women than in men [81, 83]. The median survival is now 6 years, and 1-year survival rates are up to 90%, but survival is paradoxically worse in men with PAH [84, 85]. Fourteen PAH-specific therapies that target four relevant molecular pathways (voltage gated, L-type calcium channels, nitric oxide/cGMP, endothelin, and prostacyclin) are available for PAH [81, 86]. However, current therapies for PAH improve the quality of life but do not decrease the mortality of the patients [81, 87]. Thus, a better understanding of PAH pathogenesis contributes to the identification of new targets for therapy. The pathological features of PAH include augmented vasoconstriction, vascular obstruction, vascular stiffening, endothelial dysfunction, inflammation, fibrosis, and right ventricular failure [88, 89]. Mechanisms that drive pathological vascular remodeling in the lungs of patients with PAH include cellular, genetic, and epigenetic changes. Published studies have largely focused on the role of the genetic component in the development of PAH, and the most common genetic mechanism is mutation in bone morphogenetic protein receptor 2 (BMPR2) [7], while the means of epigenetic alterations such as DNA methylation, noncoding RNAs, and histone methylation and acetylation in PAH are currently receiving increasing attention [89].

Excessive proliferation and resistance to apoptosis of the pulmonary artery smooth muscle cells (PASMCs) contribute to the reduction in arterial compliance and increased vascular resistance and blood pressure in PAH patients [89]. Therefore, maintaining homeostasis of PASMCs is critical for the prevention and treatment of PAH. Several studies have demonstrated that histone methylation plays a vital role in PASMCs and PAH [90–92]. Histone lysine methyltransferase G9a is a key enzyme for generating H3K9me2, which is an epigenetic mark of gene suppression [93]. BIX-01294, a specific inhibitor of G9a, inhibited the proliferation of fetal PASMCs and led to cell cycle arrest in the G1 phase by inducing p21 expression. In addition, the migration and contractility of fetal PASMCs were also suppressed by BIX-01294 [90]. In a hypoxia-induced PAH mouse model, *Ezh2* protein expression was positively correlated with an increase in right ventricular systolic pressure and right ventricular hypertrophy. More importantly, overexpression of *Ezh2* enhanced the proliferation and migration, but reduced the apoptosis, of human PASMCs to a greater extent than GFP transfection [91]. Using a transverse aortic constriction (TAC)-induced PAH mouse model, Shi et al. also demonstrated that *Ezh2* expression levels increased in PAH mice compared with the levels in the sham control mice, and this increase was accompanied by ROS deposition [92]. Furthermore, EPZ005687, a selective inhibitor of *Ezh2*, significantly inhibited the development of TAC-induced PAH by suppressing oxidative stress in the lung [92].

Tremendous advances have been made in elucidating the epigenetic mechanisms of PAH, but the importance of histone methylation on PAH has only recently been appreciated by researchers (Fig. 4). Studies of *G9a* and *Ezh2* on PAH indicated that histone methylation plays an essential role in PASMC proliferation and PAH. More importantly, many inhibitors targeting protein methyltransferases or demethylases have been developed, and some of them have been used in clinical trials for treating cancer or other diseases, for example, a phase II multicenter clinical trial of tazemetostat (inhibitor of EZH2) for adult subjects with INI1-negative tumors or relapsed/refractory synovial sarcoma is in the recruiting phase (ClinicalTrials.gov Identifier: NCT02601950). Therefore, further clarifying the role and molecular mechanisms of histone methylation on PAH will likely accelerate the application of inhibitors of protein methyltransferases or demethylases in the treatment of PAH. Unfortunately, despite recent advances in epigenetics, the identification of clinical epigenetic-based therapies, especially those targeting histone methylation with effective reversibility, or a cure for PAH remains a challenge for future research.

## Histone methylation in diabetic angiopathy

**Fig. 5:**
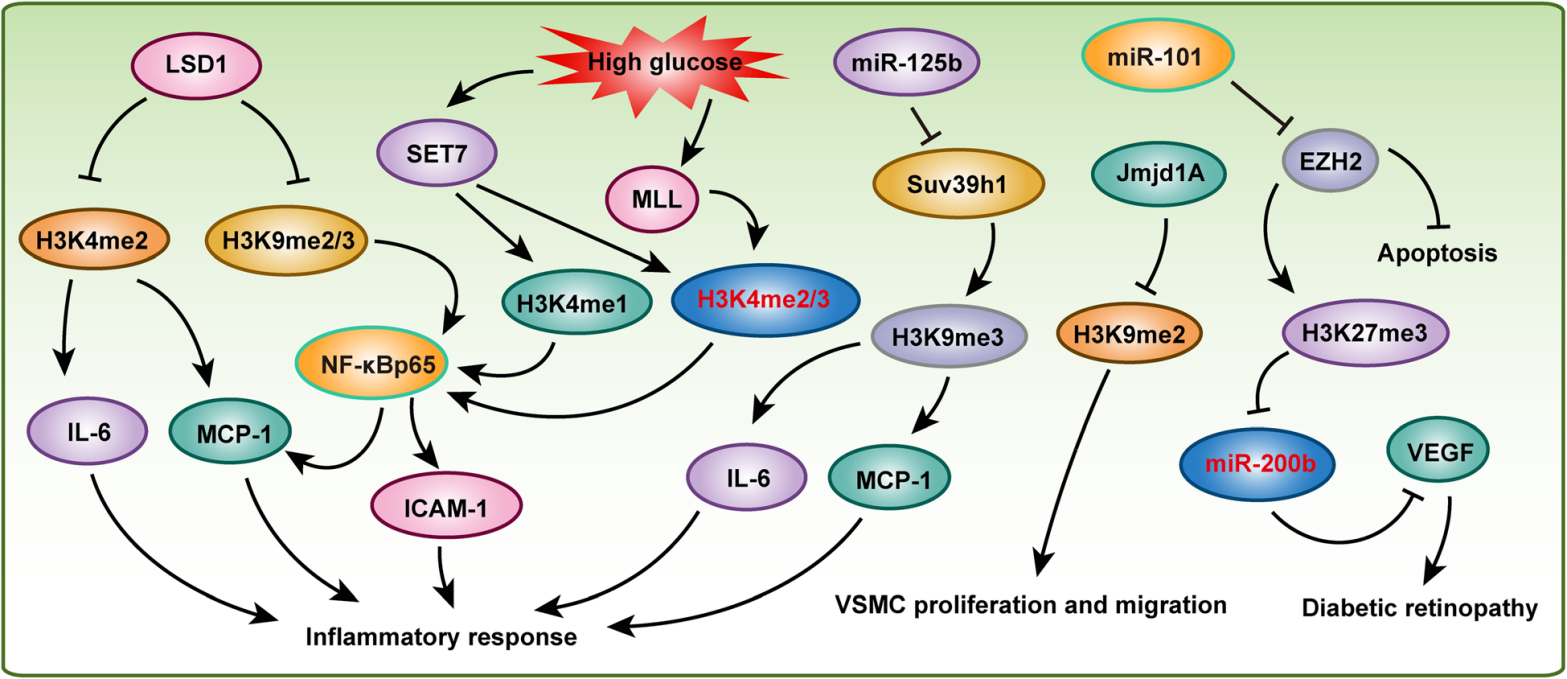
Histone methylation participates in diabetic angiopathy. Histone demethylase LSD1 and histone methyltransferases SET7, MLL, and Suv39h1 play critical roles in regulating vascular inflammatory response associated to diabetes mellitus. Jmjd1A and EZH2 are involved in VSMC proliferation, migration, or apoptosis respectively

Vascular disorders, one of the main complications of diabetes mellitus, constitute the leading cause of morbidity and mortality in patients with diabetes mellitus [94]. Interestingly, the vascular complications often persist and may progress despite improved glucose control, possibly as a result of prior episodes of hyperglycemia, in a process typically referred to as either “hyperglycemic memory” or the legacy effect [95–97]. This poorly understood “hyperglycemic memory” phenomenon poses major challenges in treating diabetes. Recent studies have demonstrated a link between epigenetic changes such as chromatin histone lysine methylation and vascular complications of diabetes (Fig. 5).

Compelling data have shown that a high glucose-induced inflammatory process plays an important role in diabetes and cardiovascular diseases [98–100]. NF-κB signaling is one of the most important pathways regulating inflammation via initiating inflammatory factors and cytokine expression. Transient hyperglycemia stimulation induced sustained upregulation of the NF-κBp65 gene, which is associated with increased H3K4me1 and decreased H3K9me2 and H3K9me3 on the NF-κBp65 promoter in aortic endothelial cells [97]. Histone methyltransferases SET7 and LSD1 mediated H3K4 mono-methylation and H3K9me2/3 demethylation, respectively [97]. Moreover, increased NF-κBp65 significantly promoted inflammatory factor monocyte chemoattractant protein-1 (MCP-1) expression [97]. Han et al. also demonstrated that, in EA.hy926 (a human umbilical vein cell line) cells treated with high glucose, H3K4me2 and H3K4me3 marks were enriched on the promotor of the MCP-1 gene [101]. Furthermore, they found that the histone methyltransferases MLL and SET7, which catalyze H3K4 methylation, were increased on the MCP-1 promotor, while the demethylase LSD1 was decreased in endothelial cells challenged with high glucose [101]. In peripheral blood monocytes (PBMs) isolated from 44 T2DM patients and 24 age-matched controls, the T2DM patients showed higher SET7 expression levels than were shown by the controls, and SET7 methylated H3K4me1 on the promoter of NF-κBp65 to accelerate its expression, resulting in ICAM-1 and MCP-1 secretion into plasma to induce oxidative stress and the inflammatory response [102]. Similarly, in human aortic endothelial cells (HAECs), knockdown of SET7 reduced H3K4me1 mark and abolished NF-kB-dependent oxidant and inflammatory signaling [102]. These studies indicated that SET7 plays a pivotal role in glucose-mediated inflammatory response and is therefore a candidate gene for the induction of diabetic vascular complications. In addition, *Lsd1*, which demethylates H3K4, was significantly decreased in *db*/*db* mice compared with the level in their counterparts, while H3K4me2 was elevated at the promoters of the inflammatory genes *Mcp*-*1* and *Il*-*6* in *db*/*db* VSMCs. Silencing of *Lsd1* facilitated inflammatory gene expression and enhanced VSMC-monocyte binding in nondiabetic VSMCs. In contrast, overexpression of *Lsd1* inhibited these effects [103]. NADPH oxidase 4 (Nox4) and eNOS, which are important enzymatic sources of reactive oxygen species (ROS) in diabetic vasculature, were regulated by H3K4me1, H3K9me2, and H3K9me3 resulting in endothelial dysfunction [104].

As H3K9 methylation levels are elevated upon high glucose stimulation, its methyltransferases *Suv39h1*/*2* were also reported to be involved in vascular complications of diabetes [105, 106]. For example, in vascular smooth muscle cells (MVSMCs) from type 2 diabetic *db*/*db* mice, miR-125b, which targets *Suv39h1*, was upregulated, while the *Suv39h1* protein level was lower than that in the *db/+* controls [105]. Knocking down *Suv39h1* in normal human VSMCs increased inflammatory gene expression by decreasing H3K9me3 occupancy at its promoter. In contrast, overexpression of *Suv39h1* in *db*/*db* VSMCs reversed this diabetic phenotype [106]. Furthermore, miR-125b mimics increased the expression of the inflammatory genes *Mcp*-*1* and *Il*-*6* by targeting *Suv39h1* to reduce H3K9me3 mark at their promoters in nondiabetic cells [105]. In addition, the minor T allele of the exonic SNP rs17353856 in Suv39h2 (a member of the Suv39h1 family) was associated with diabetic retinopathy and cardiovascular disease in the FinnDiane cohort [107]. JMJD1A is the demethylase of H3K9me2, and H3K9me2 decreases when JMJD1A is elevated in diabetic vessels [108]. *Jmjd1a* promoted high glucose and Ang II-induced proliferation and migration of VSMCs. Moreover, *Jmjd1a* overexpression accelerated balloon injury-induced neointima formation in diabetic rats in which glucose was not controlled, and this effect was mediated by the Rho/ROCK and Ang II/AGTR1 pathways [108]. Interestingly, in brown adipocytes, *Jmjd1a* was phosphorylated at S265 by protein kinase A (PKA) to increase its interaction with the SWI/SNF nucleosome remodeling complex and DNA-bound *Pparγ*, thereby activating the β1-adrenergic receptor gene (*Adrb1*) and its downstream targets, including *Ucp1*. Unexpectedly, this rapid gene induction was found to be dependent on S265 phosphorylation of *Jmjd1a* but not on its demethylation activity [109].

H3K27me3 methylated by Polycomb repressive complex 2 (PRC2) is one of the most widely studied histone marks. In human retinal microvascular endothelial cells, PRC2 methylated H3K27me3 to inhibit miR-200b which targeted to vascular endothelial growth factor (VEGF) under high glucose conditions. Increased VEGF increased ocular permeability and neovascularization and accelerated the development of diabetic retinopathy [110]. EZH2 is the main active subunit of PRC2 that initiates and maintains H3K27me3. In human fetal endothelial cells (ECs) of the umbilical cord vein (HUVECs) in gestational diabetes mellitus patients, miR-101 was upregulated, leading to H3K27me3 downregulation by targeting EZH2 [111]. Interestingly, both gestational diabetes mellitus and high glucose could reduce EZH2 binding to the miR-101 locus in HUVECs, and EZH2 overexpression decreased the relative apoptotic activity and increased the migratory capacity of the HUVECs exposed to gestational diabetes mellitus [111]. These results indicate that EZH2-miR-101 creates a positive feedback loop that regulates endothelial cell dysfunction in gestational diabetes mellitus.

## Histone methylation in endothelial cell dysfunction

**Fig. 6:**
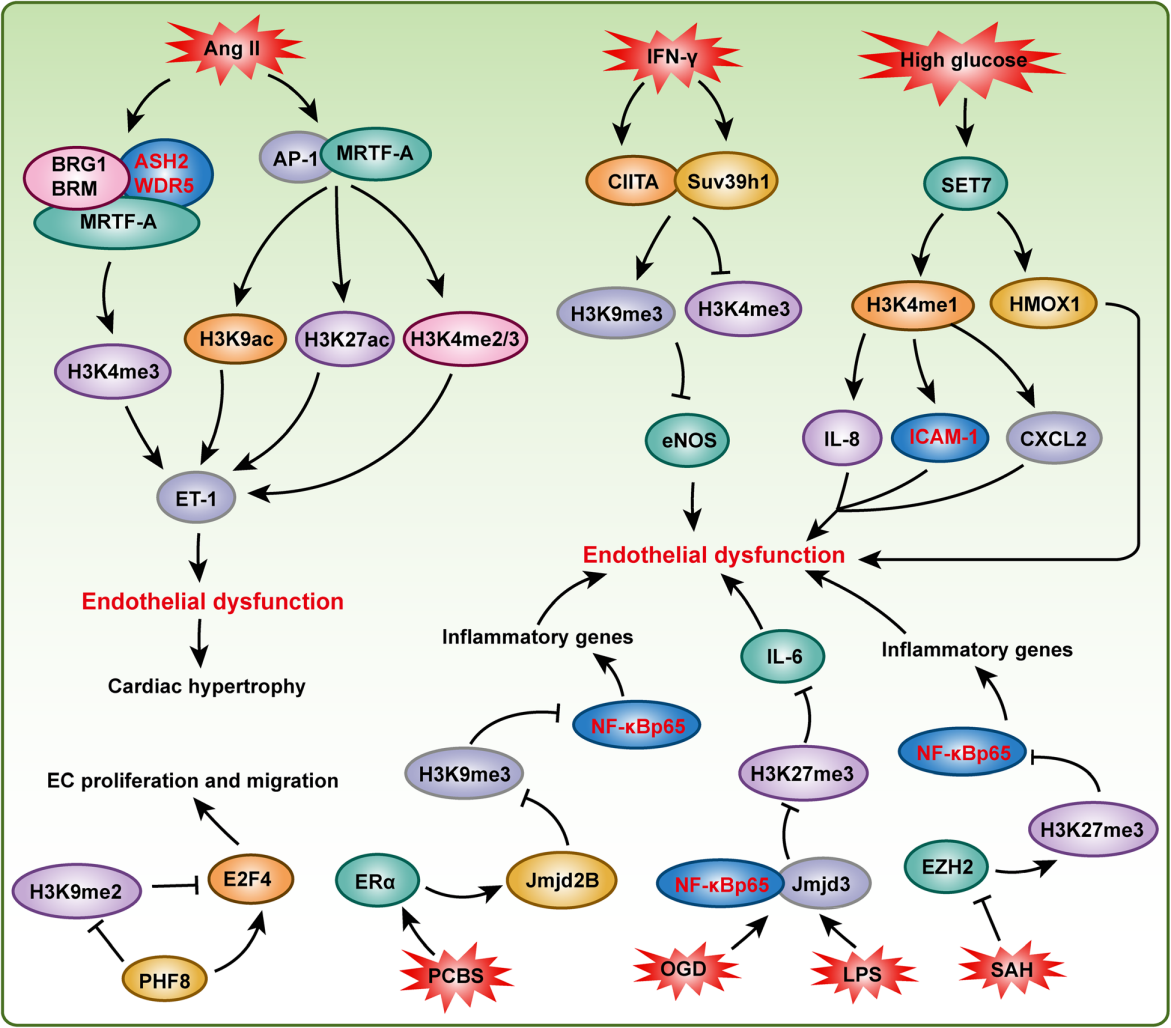
Histone methylation is important for maintaining endothelial cell homeostasis. Under stimulation of many stresses, such as IFN-γ, LPS, and high glucose, the function of endothelial cells were regulated by many histone methyltransferases (e.g., Suv39h1, SET7, and EZH2) and demethylases (e.g., Jmjd2B, Jmjd3, and PHF8)

Vascular EC dysfunction is one of the major causes of cardiovascular disease, such as hypertension, cardiac remodeling, and diabetic cardiomyopathy. Epigenetic mechanisms, especially histone methylation, play essential roles in regulating the function of ECs and their homeostasis (Fig. 6). eNOS is constitutively expressed in ECs, and it plays a critical role in vascular wall homeostasis and the regulation of vasomotor tone. Thus, clarifying the mechanisms regulating eNOS expression in ECs is essential to understand the way these mechanisms may be perturbed in vascular biology. The expression level of eNOS is reduced when ECs are treated with IFN-γ, and the complex formed by class II trans-activator (CIITA) and Suv39h1 directly binds to the proximal eNOS promoter to repress transcription, and H3K9me3, which is induced by Suv39h1, mediates IFN-γ-induced eNOS repression [112]. In addition to methylated H3K9, H3K9ac, H4K12ac, H3K4me2, and H3K4me3 also participate in the regulation of eNOS expression in ECs [77]. In contrast to eNOS, endothelin (ET-1) is clearly the most potent vasoconstrictor. In response to Ang II stimulation, myocardin-related transcription factor A (MRTF-A) is recruited to the ET-1 promoter by c-Jun/c-Fos (AP-1), which alters the chromatin structure by modulating H3K9ac, H3K27ac, and H3K4me2/3 on the ET-1 promoter [113]. Further investigation indicated that the *Brg1*/*Brm* and *Ash2*/*Wdr5* complexes are recruited by MRTF-A to catalyze H3K4 methylation on the ET-1 promoter, which induces ET-1 transactivation in ECs to accelerate Ang II-induced cardiac hypertrophy and fibrosis [114].

High levels of glucose have been found to result in pathophysiological changes of vascular cells, contributing to accelerated atherosclerosis and other vascular complications associated with diabetes, and epigenetic changes have been implicated in the persisting vascular effects of hyperglycemia [115]. For example, in response to hyperglycemia, histone methyltransferase *Setd7* protein accumulates in the nucleus of ECs, which promotes *Il*-*8*, *Icam1*, and *Cxcl2* expression in an H3K4me1-dependent manner, and inhibits *Hmox1* expression in an H3K4me1-independent fashion to regulate “hyperglycemic memory” [115]. In ECs with oxygen-glucose deprivation/reperfusion injury, histone H3K27me3 demethylase *Jmjd3* expression is upregulated, and the increase in *Jmjd3* leads to greater *Jmjd3* interactions with *Nf*-*κb* (p65/p50) and CCAAT-enhancer-binding protein β at the *Il*-*6* gene promoter, which decreases H3K27me3 levels to promote *Il*-*6* expression to regulate the inflammatory response [116]. Similarly, LPS treatment promotes *Jmjd3* expression in ECs to activate the expression of target genes by synergizing with *Nf*-*κb* and demethylation of H3K27me3 [117]. *Ezh2*, the methyltransferase that targets H3K27, was suppressed by excess S-adenosylhomocysteine (SAH) in the ECs, and decreased *Ezh2* contributes to *Nf*-*κb* activation and the consequent vascular inflammatory response [118]. Environmental pollutants were reported to increase the incidence rates of cardiovascular diseases, while the underlying epigenetic mechanisms were largely unknown. Liu et al. treated ECs with polychlorinated biphenyls (PCBs), which are common environmental pollutants, and the coplanar PCBs induced not only *Nf*-*κb* signaling and *Nf*-*κb* target inflammatory gene activation but also histone H3K9me3 demethylase jumonji domain-containing protein 2B (*Jmjd2b*) expression. The increased accumulation of *Jmjd2b* on the p65 promoter led to the demethylation of the H3K9me3 repression mark and to the observed upregulation of p65 and associated inflammatory genes [119]. Another demethylase, histone plant homeodomain finger protein 8 (PHF8), catalyzed the removal of methyl groups from H3K9 and H4K20. In ECs, PHF8 maintained E2F4 expression by demethylating H3K9me2 at the E2F4 transcriptional start site to facilitate endothelial cell proliferation, survival, and the capacity for migration and development of capillary-like structures [120]. G9a is the methyltransferase that targets H3K9, and inhibition of G9a activity by BIX-01294 or knockdown by shRNA attenuates the proliferation of human microvascular ECs, and arresting them in the G1 phase of the cell cycle by regulating the phosphorylation of CHK1 [121]. In addition, histone methyltransferase MLL contributes to endothelial-cell sprout formation by regulating HoxA9 and EphB4 expression [122].

## Histone methylation in tumor angiogenesis

It is well known that angiogenesis is a main contributor to tumor growth and the metastatic process. Therefore, approximately half a century ago, some scholars proposed the concept of inhibiting tumor angiogenesis for treating solid tumors. The anti-angiogenic drugs were expected to decrease or even block the oxygen and nutritional supply of tumor and then to arrest tumor growth, and displayed minimal toxic side effects to healthy tissues at the same time. Given that VEGFA is the most important regulator of tumor angiogenesis, Bevacizumab (Avastin), a humanized monoclonal anti-VEGFA antibody, is a typical example of anti-tumor angiogenesis and it is now used as anti-angiogenic drug in several forms of cancers, including breast, colorectal, and lung cancers [123]. Thus, the mechanisms that regulates the expression or activating of VEGFA are critical for regulating tumor angiogenesis. Importantly, histone methylation and its responsible methyltransferases or demethylases are indispensable for VEGFA and its receptors regulation and tumor angiogenesis.

It is reported that histone methyltransferase *Dot1l* deletion results in embryonic lethality and cardiovascular defects including decreased vasculature [47]. In HUVECs, knockdown of DOT1L results in decreased cell viability, migration, tube formation, and capillary sprout formation, as well as reduced formation of functional vascular networks in vivo, which was mediated by H3K79me2 and cooperating with transcription factor ETS-1 to regulate VEGFR2 expression [124]. In breast cancer patients, histone methyltransferase SET7 and transcription factor GATA1 expression levels were upregulated and positively correlated with VEGFA expression and microvessel number. Furthermore, SET7 associates with GATA1 to promote VEGFA transcription and breast tumor angiogenesis [125]. However, by using ProtoArray system, Cohn et al. identified 172 new SETD3 interacting proteins, and further investigation found that SETD3 binds and methylates the transcription factor FoxM1 to inhibit VEGFA expression under hypoxia [126]. In addition, GSK126, an EZH2 inhibitor, inhibits gastric cancer and lung adenocarcinoma cell migration and angiogenesis in solid tumor cell lines through downregulation of VEGFA expression [127]. In addition to the regulation of VEGFA or its receptor expression, HMTs also regulate PTMs of VEGFR1 or alternative splicing of VEGFA to affect tumor angiogenesis. For example, histone methyltransferase SMYD3 expression level was elevated in colorectal, hepatocellular, and breast carcinomas, and elevated SMYD3 interacts with VEGFR1 to methylate VEGFR1 at its lysine 831. Furthermore, methylation of VEGFR1 enhanced its kinase activity in cells [128]. The H3K9 methyltransferase G9a was reported to regulate the alternative splicing of VEGFA (exclusion of VEGFA exon 6a) via interacting with chromatin modulator HP1γ and methylated H3K9 to recruit splicing regulator SRSF1, but this kind of alternative splicing did not alter total VEGFA mRNA levels [129].

HIF1α is another key regulator of tumor growth and angiogenesis as a transcriptional regulator of VEGFA [130]. The stability and function of the HIF1α protein are also affected by methylation. BIX01294, a G9a-specific inhibitor, decreased expression levels of HIF1α, VEGFA, proline hydroxylase 2 (PHD2), hydroxylated HIF1α and von Hippel-Lindau protein (pVHL), as well as shortened the half-life of HIF1α in HepG2 human hepatocellular carcinoma cells under hypoxic conditions. Furthermore, BIX01294 suppressed VEGFA-induced MMP2 activity and phosphorylation of VEGFR2, focal adhesion kinase (FAK), and paxillin in HUVECs [131]. These results indicated that histone methyltransferase G9a could facilitate HIF1α stability and VEGFA-induced angiogenesis. In prostate cancer, elevated expression of LSD1 correlates with prostate cancer recurrence and with increased VEGFA expression, and knockdown of LSD1 in prostate cancer cells decreases VEGFA expression [132]. Importantly, LSD1 demethylates HIF1α at lysine 391 to protect HIF1α against ubiquitin-mediated protein degradation. HIF1α stabilized by LSD1 cooperates with CBP and MTA1 to enhance VEGFA-induced tumor angiogenesis [130].

**Fig. 7:**
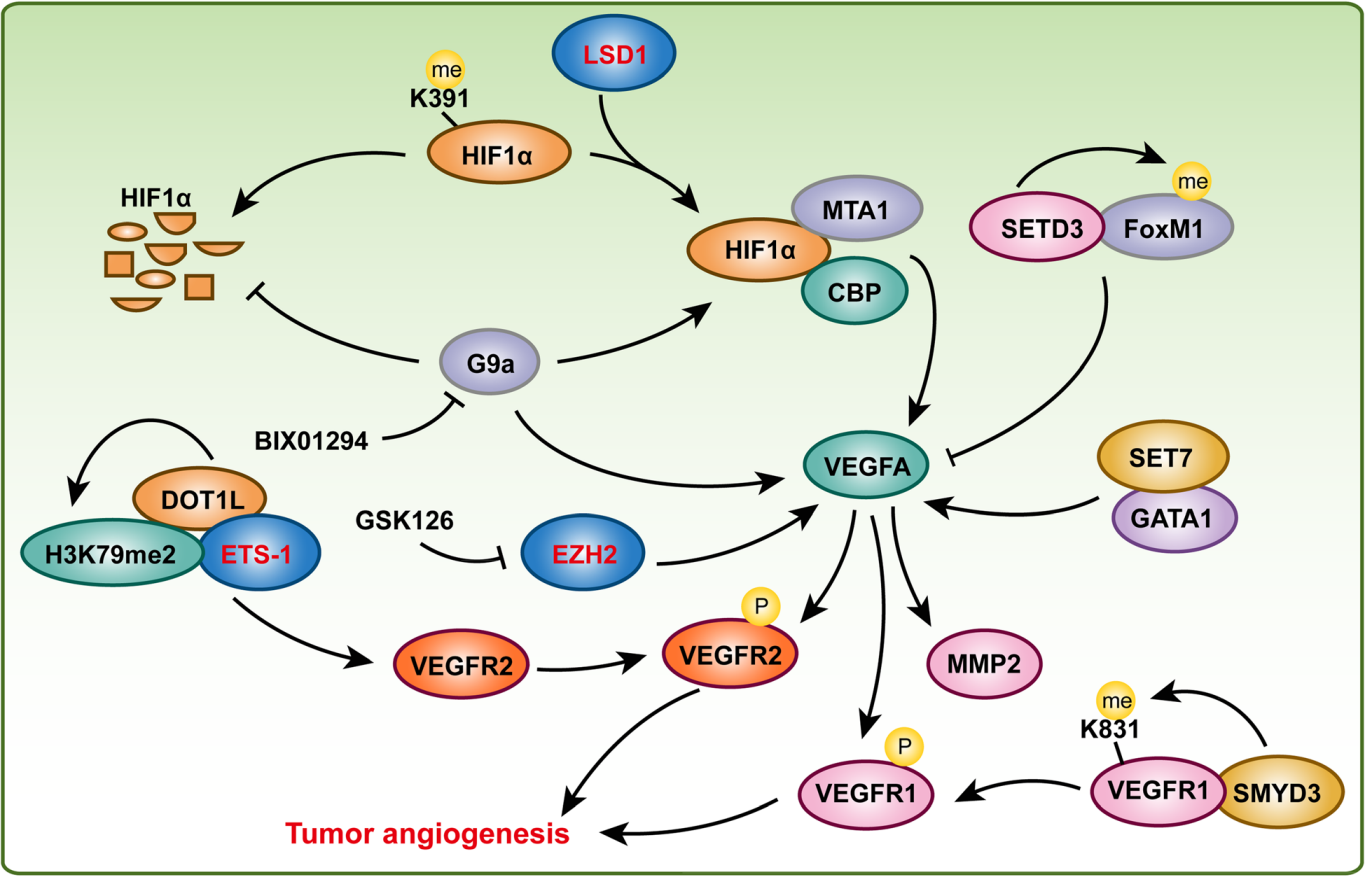
Histone methyltransferases and demethylases are involved in tumor angiogenesis. HIF signaling pathway and VEGFA signaling pathway play a central role in tumor angiogenesis. LSD1 and G9a could promote HIF1α expression and increase its stability, which subsequently accelerates VEGFA and its downstream genes expression, and activates VEGFA signaling pathway to regulate tumor angiogenesis. In addition, EZH2, DOT1L, SMYD3, SETD3 and SET7 are also involved in regulating VEGFA expression or VEGFA signaling pathway during tumor angiogenesis

These studies indicated that HMTs and HDMTs not only regulate VEGFA and HIF1α expression but also involve in their PTMs, activity, and stability to affect tumor angiogenesis (Fig. 7).

## Histone methylation in other forms of vasculopathy

In addition to the aforementioned vascular diseases, histone methylation is also involved in other forms of vasculopathy. For example, Chen et al. reported that, in indoxyl sulfate-induced VSMCs, the characteristics of osteoblastic differentiation and calcification are manifested with the downregulation of the expression of histone methyltransferase *Set7*/*9* and with autophagy activation, which indicates that *Set7*/*9* downregulation and autophagy activation may be the key mechanisms of indoxyl sulfate-induced vascular calcification in chronic kidney disease [133]. Intercellular adhesion molecule 1 (*Icam1*) mediates the adhesion and transmigration of leukocytes across the endothelium to promote inflammation in the vasculature. In human brain microvascular endothelial cells and mouse brain microvessels, the pro-inflammatory cytokine *Tnf*-*α* dramatically increases *Icam1* mRNA and protein levels by regulating H3K9me2, which is achieved by treatments with histone methyltransferase *G9a* and demethylase *Kdm4b*. Moreover, *G9a* overexpression or *Icam1* or *Kdm4b* depletion reduces inflammation-induced leukocyte extravasation, which indicates that blocking *Icam1* or *Kdm4b* may offer a novel therapeutic approach for treating brain diseases [134]. Anti-neutrophil cytoplasmic autoantibody-associated vasculitis (AAV) is a systemic autoimmune disease characterized by destructive vascular inflammation, which is associated with autoantibodies directed against the neutrophil granule proteins myeloperoxidase (MPO) or proteinase 3 (PR3). H3K9 methylation and its corresponding methyltransferases EHMT1 and EHMT2 were depleted most extensively at the MPO and PR3 genes, while H3K4 methylation and H4K16 acetylation were enriched at the MPO and PR3 genes in patients with active disease [135]. In addition, Karnewar et al. demonstrated that H3K79me was involved in metformin-regulated mitochondrial biogenesis and senescence in age-associated vascular dysfunction [136].

## Conclusion and perspective

In this review, we highlight the role of histone methylation in the vascular development and vascular-related diseases, such as aortic dissection and pulmonary arterial hypertension. Currently, our understanding of histone methylation in vascular biology is rudimentary, but the observations presented in this review offer a broad base for further discovery. Although great progress has been made in the field of histone methylation in vascular biology, it is important to raise a few points. First, the published studies primarily focused on a few molecules related to histone methylation, such as EZH2, G9a, and LSD1, but did not clarify the roles of other HMTs and HDMTs. Second, few nonhistone targets that mediate the function of HMTs and HDMTs in vascular biology have been identified; however, nonhistone proteins are commonly methylated by HMTs in other biological processes (e.g., cancer). Methylation of the nonhistone protein not only affects protein activity and stability but also interacts with other posttranslational modifications to regulate its function; therefore, the discovery of more methylation signaling pathways in vascular biology is important. Third, do HMTs or HDMTs function in vascular biology independent of their methyltransferase or demethylase activity? Fourth, more conditional knockout animal models rather than global knockout models should be used to investigate the roles and mechanisms of HMTs and HDMTs in vascular biology in the future. Fifth, S-adenosylmethionine (SAM), the methyl-donating substrate of histone methyltransferases, and S-adenosylhomocysteine (SAH) link one-carbon metabolism to methylation status. Extensive research demonstrated that one carbon metabolism is closely related to histone methylation, and they play critical roles in embryonic development, cancer, and neurodegenerative diseases. However, there is almost no study published that tried to investigate how one carbon metabolism works together with histone methylation to affect vascular biology or diseases. Thus, more efforts should be pained to delve into this new field, which may open new pathways for pharmacological intervention in vascular diseases. Sixth, some inhibitors of HMTs or HDMTs may have the potential to reverse pathological vascular changes, and more attention should be paid to the clinical application of these inhibitors. We suspect that inhibitors of HMTs and HDMTs have great potential to remedy vascular-related diseases. Nevertheless, although more of these inhibitors are likely to be developed, the issue of specificity may be a limiting factor for their safe and efficacious widespread use.

## Abbreviations

AAA: Abdominal aortic aneurysm
AAD: Acute aortic dissection
AAS: Acute thoracic aortic syndromes
AAV: Anti-neutrophil cytoplasmic autoantibody-associated vasculitis
ABCA1: ATP-binding cassette transporter A1
ACE1: Angiotensin-converting enzyme 1
AD: Aortic dissection
ADMA: Asymmetric dimethylarginine
AdoMet: S-Adenosyl-l-methionine
Adrb1: β1-adrenergic receptor gene
ANP: Atrial natriuretic peptide
AP1: Activating protein 1
BMPR2: Bone morphogenetic protein receptor 2
BNP: Brain natriuretic peptide
cGMP: Cyclic guanosine monophosphate
DNMT1: DNA methyltransferase 1
ECM: Extracellular matrix
ECs: Endothelial cells
eNOS: Endothelial NO synthase
ET-1: Endothelin
HAECs: Human aortic endothelial cells
HDMTs: Histone demethylases
HHcy: Hyperhomocysteinemia
HMT: Histone methyltransferase
HSD11B2: Hydroxysteroid dehydrogenase-11β2 enzyme
ICAM1: Intercellular adhesion molecule 1
IMH: Intramural hematoma
JMJD2B: Jumonji domain-containing protein 2B
LDL: Low-density lipoprotein
LRP6: Low-density lipoprotein receptor-related protein 6
LSD1: Lysine-specific demethylase-1
MCP-1: Monocyte chemoattractant protein-1
MeCP2: Methyl CpG-binding protein-2
MEF2: Myocyte enhancing factor-2
MMA: Monomethylation
mPAP: Mean pulmonary artery pressure
MPO: Myeloperoxidase
MRTF-A: Myocardin-related transcription factor A
Nox4: NADPH oxidase 4
PAH: Pulmonary arterial hypertension
PASMCs: Pulmonary artery smooth muscle cells
PAU: Penetrating aortic ulcer
PBM: Peripheral blood monocytes
PCBs: Polychlorinated biphenyls
PCWP: Pulmonary capillary wedge pressure
PH: Pulmonary hypertension
PHF8: Plant homeodomain finger protein 8
PKA: Protein kinase A
PKMTs: Protein lysine methyltransferases
PlGF: Placental growth factor
PR3: Proteinase 3
PRC2: Polycomb repressive complex 2
PRMTs: Protein arginine methyltransferases
RAAS: Renin-angiotensin-aldosterone system
ROS: Reactive oxygen species
SAH: S-adenosylhomocysteine
SDMA: Symmetric dimethylarginine
SET: Suppressor of variegation, enhancer of Zeste, Trithorax
SET1: Suv, Ez, and Trithorax domain 1
SHR: Spontaneously hypertensive rat
SMCs: Smooth muscle cells
TAAs: Thoracic aortic aneurysms
TAC: Transverse aortic constriction
VEGF: Vascular endothelial growth factor
